# Notes and Formulæ

**Published:** 1889-06

**Authors:** 


### PRACTICAL NOTES AND FORMULÆ.

**For Dysentery in Children.**—

℞ Castor oil, . . . . . . . . ʒi.

Pulv. accaceæ, . . . . . . . ʒi.

Paregoric, . . . . . . . . ʒss

Mint water, . . . . . . . . ℥ij

M. Teaspoonful every two to three hours in a child six to twelve months old.

**For Dysentery in the Adult.**—

℞ Calomel, . . . . . . . . grs. x.
Dover's powder, . . . . . . grs. v.

M. In two hours after taking the above commence with the following:

℞ Epsom salts, . . . . . . . ℥i.

Water, . . . . . . . . ½ pint.

M. Take tablespoonful every hour until free evacuations have been produced, then put a large mustard plaster to the abdomen and give the following:

℞ Muriate of morphine, . . . . . grs. ii.
Pulv. ipecac.
Monobrominated camphor, . . . ää grs. iv.

M. Divide into eight powders. Take one every three hours until discharges are three hours apart, then one after each evacuation until relieved.

**To Abort Bronchitis.**—Dr. H. C. Wood, according to the *Journal of the American Medical Association*, recommends the following:

℞ Potass. citrate, . . . . . . . ℥j

Syr. ipecac, . . . . . . . . ℥j

Succi limonis, . . . . . . . ℥ij

Aquæ, . . . . . . . . . ℥iij

M. Ft. Sol.

Sig.: Two teaspoonfuls every two hours.

**Gargle to Prevent Loosening of the Teeth.**—By Dr. Quincerat, France.

℞ Acid tanic, . . . . . . . . ʒij.
Tinct. iodi., . . . . . . . . gtt. 75.
Potass. iodi., . . . . . . . gr. 16.
Tinct. myrrhæ, . . . . . . . gtt. 75.
Aquæ, . . . . . . . . . ℥6½.

M. Sig.: A tablespoonful in a third of a glass of warm water to bathe the gums with.—*Leonard's Med. Jour.*

**Cough Mixture.**—By Prof. Atkinson, Philadelphia, Pa.

| | | |
|---|---|---|
| ℞ | Ammonii muriatis, . . . . . . | ʒij. |
| | Syrupi senegæ, . . . . . . . | ℥ss. |
| | Misturæ glycyrrhizæ comp., . . . | ℥iv. |

M. Sig.: ʒj every three hours.—*Ex.*

**Dysmenorrhœa.**—By Z. T. Housman, Balrdstown, O.

| | | |
|---|---|---|
| ~ | Fl. ex. cimcifuga vac., . . . , . | ℥ij. |
| | Fl. ex. gelsemium, sempervirens, (green root), | ℥j. |

M. Sig.: Ten drops every two or three hours. Begin treatment one day prior to expected menstruation.

**For Eczema of the Anus and Genitals.**—

| | | |
|---|---|---|
| ℞. | Oleate of Cocaine . . . . | 1 part. |
| | Olive Oil . . . . . . | 2 parts. |
| | Lanolin . . . . . . . | 10 parts. |

M. Sig.: Apply this ointment twice a day to the affected part.—*Ib.*

**Itch.**—Dr. Greulich claims that the following never fails him:

| | | |
|---|---|---|
| ℞. | Resorcin . . . . | 1 drachm. |
| | Vaseline . . . . | 1 ounce. |

M. Sig.: Apply every night at bed time.—*Ib.*

**Ulceration of the Ear.**—A case of chronic ulceration of the ear, emitting a fetid discharge, was treated by the following:

| | | |
|---|---|---|
| ℞ | Acid. nitric, | 10 drops. |
| | Aquæ, . . | 8 ounces. |

A teaspoonful of this mixture thrown into the ear two or three times a day, put a stop to the disease in less than a week.—*Ib.*

**For Dysentery of Children.**—

| | | |
|---|---|---|
| ℞ | Castor oil, . . . . . . . . | ℥ss. |
| | Aromatic syr. rhubarb, . . . . | ℥ij. |
| | Paregoric, . . . . . . . . | ʒi. |

M. Shake and give a teaspoonful every three hours to a child one year old.

**For Dysentery in Teething Children.**—

| | | |
|---|---|---|
| ℞ | Aconite, . . . . . . . | 4 drops. |
| | Water, . . . . . . . | 43 ounces. |
| | Chloranodyne (P. D. & Co.) . . | 10 drops. |

M. Teaspoonful every hour to a child one year old in dysentery attended with fever, first giving a dose of castor oil to remove offensive matter from the bowels.

